# Incidence, risk factors and outcome of acute kidney injury in critically ill COVID-19 patients in Tyrol, Austria: a prospective multicenter registry study

**DOI:** 10.1007/s40620-023-01760-3

**Published:** 2023-10-14

**Authors:** Timo Mayerhöfer, Fabian Perschinka, Sebastian J. Klein, Andreas Peer, Georg F. Lehner, Romuald Bellmann, Lukas Gasteiger, Markus Mittermayr, Robert Breitkopf, Stephan Eschertzhuber, Simon Mathis, Anna Fiala, Dietmar Fries, Mathias Ströhle, Eva Foidl, Walter Hasibeder, Raimund Helbok, Lukas Kirchmair, Birgit Stögermüller, Christoph Krismer, Tatjana Heiner, Eugen Ladner, Claudius Thomé, Christian Preuß-Hernandez, Andreas Mayr, Miriam Potocnik, Bruno Reitter, Jürgen Brunner, Stefanie Zagitzer-Hofer, Alexandra Ribitsch, Michael Joannidis

**Affiliations:** 1 Division of Intensive Care and Emergency Medicine, Department of Internal Medicine, Medical University Innsbruck, Anichstrasse 35, 6020 Innsbruck, Austria; 2 Internal Medicine II, Gastroenterology, Hepatology and Rheumatology, Karl Landsteiner University of Health Sciences, University Hospital St. Pölten, St. Pölten, Austria; 3 Department of Anesthesia and Critical Care Medicine, Medical University Innsbruck, Innsbruck, Austria; 4 Department of Anesthesia and Intensive Care Medicine, Hospital Hall, Hall, Austria; 5 Department of General and Surgical Intensive Care Medicine, Medical University Innsbruck, Innsbruck, Austria; 6 Department of Anesthesia and Intensive Care Medicine, Hospital Kufstein, Kufstein, Austria; 7 Department of Anesthesiology and Critical Care Medicine, Hospital St. Vinzenz Zams, Zams, Austria; 8 Department of Neurology, Medical University Innsbruck, Innsbruck, Austria; 9 Department of Anesthesia and Critical Care Medicine, Hospital Schwaz, Schwaz, Austria; 10 Department of Internal Medicine, Hospital St. Vinzenz Zams, Zams, Austria; 11 Department of Anesthesia and Intensive Care Medicine, Hospital Reutte, Reutte, Austria; 12 Department of Anesthesia and Intensive Care Medicine, Hospital Lienz, Lienz, Austria; 13 Department of Neurosurgery, Medical University Innsbruck, Innsbruck, Austria; 14 Department of Anesthesia and Intensive Care Medicine, Hospital St. Johann in Tyrol, St. Johann in Tyrol, Austria; 15 Department of Pediatrics, Medical University Innsbruck, Innsbruck, Austria; 16 Department of Internal Medicine, Hospital Hall, Hall, Austria; 17 Department of Internal Medicine, Hospital Lienz, Lienz, Austria; 18 Department of Neurology, Johannes Kepler University Linz, Linz, Austria; 19 Faculty of Medicine and Dentistry, Danube Private University, Krems, Austria

**Keywords:** SARS-CoV-2, Intensive Care Unit, Renal, Critical care, Pandemic

## Abstract

**Introduction:**

Acute kidney injury is a frequent complication in critically ill patients with and without COVID-19. The aim of this study was to evaluate the incidence of, and risk factors for, acute kidney injury and its effect on clinical outcomes of critically ill COVID-19 patients in Tyrol, Austria.

**Methods:**

This multicenter prospective registry study included adult patients with a SARS-CoV-2 infection confirmed by polymerase chain reaction, who were treated in one of the 12 dedicated intensive care units during the COVID-19 pandemic from February 2020 until May 2022.

**Results:**

In total, 1042 patients were included during the study period. The median age of the overall cohort was 66 years. Of the included patients, 267 (26%) developed acute kidney injury during their intensive care unit stay. In total, 12.3% (*n* = 126) required renal replacement therapy with a median duration of 9 (IQR 3–18) days. In patients with acute kidney injury the rate of invasive mechanical ventilation was significantly higher with 85% (*n* = 227) compared to 41% (*n* = 312) in the no acute kidney injury group (*p* < 0.001). The most important risk factors for acute kidney injury were invasive mechanical ventilation (OR = 4.19, *p* < 0.001), vasopressor use (*OR* = 3.17, *p* < 0.001) and chronic kidney disease (OR = 2.30, *p* < 0.001) in a multivariable logistic regression analysis. Hospital and intensive care unit mortality were significantly higher in patients with acute kidney injury compared to patients without acute kidney injury (Hospital mortality: 52.1% vs. 17.2%, *p* < 0.001, ICU-mortality: 47.2% vs. 14.7%, *p* < 0.001).

**Conclusion:**

As in non-COVID-19 patients, acute kidney injury is clearly associated with increased mortality in critically ill COVID-19 patients. Among known risk factors, invasive mechanical ventilation has been identified as an independent and strong predictor of acute kidney injury.

**Graphical abstract:**

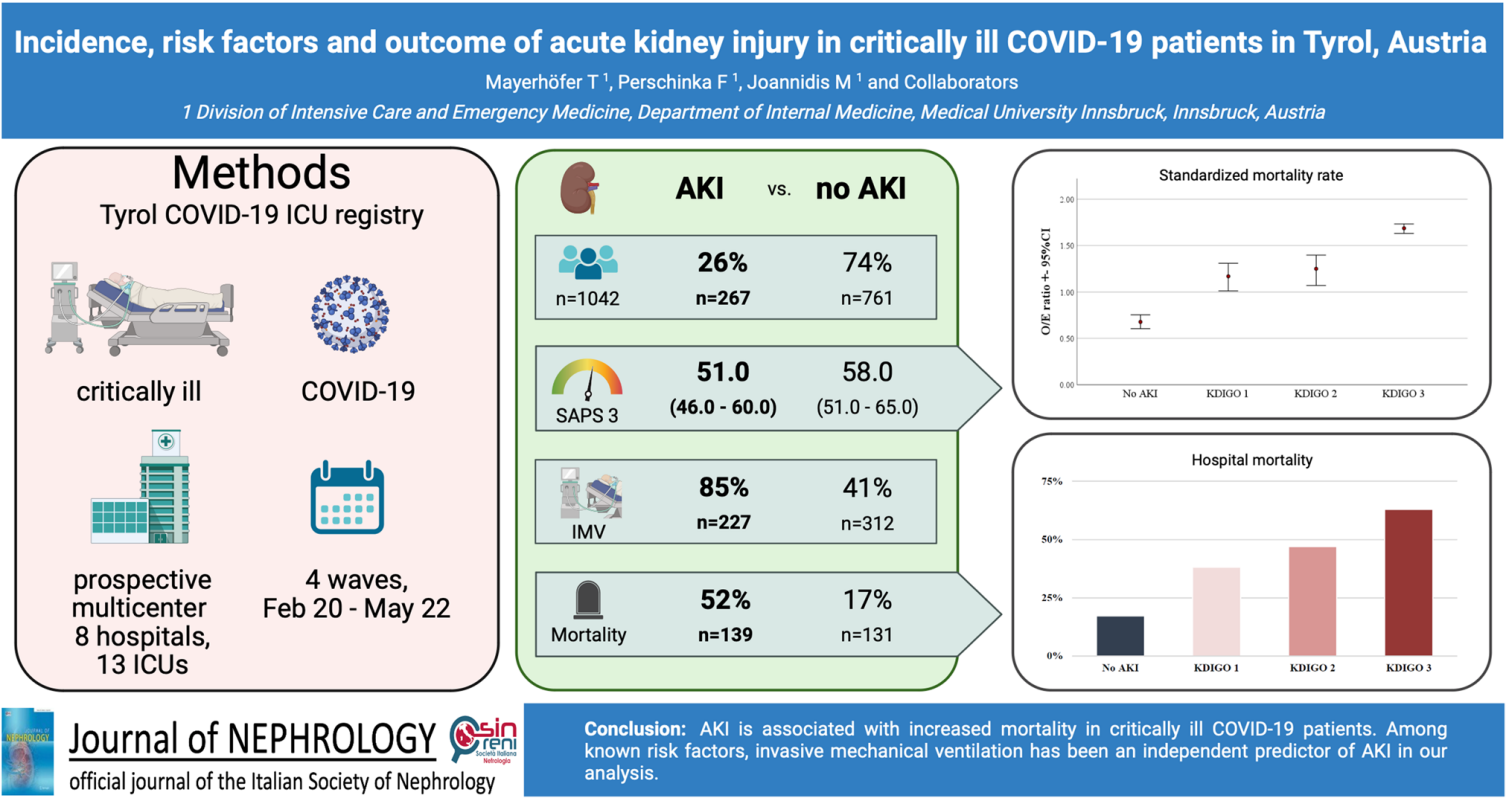

**Supplementary Information:**

The online version contains supplementary material available at 10.1007/s40620-023-01760-3.

## Introduction

The coronavirus disease 2019 (COVID-19) caused by the severe acute respiratory syndrome coronavirus 2 (SARS-CoV-2) affected intensive care units (ICUs) around the world for more than 2 years [1]. In addition to the lungs, numerous other organs including the kidneys were involved, particularly in critically ill patients [2, 3].

The incidence of acute kidney injury (AKI) in patients admitted to an ICU before the COVID-19 pandemic was up to 57.3% in the multinational AKI-EPI study [4]. Early reports of the incidence of AKI in COVID-19 patients varied widely from very low rates in China [5] to relatively high rates in Europe and the United States [6, 7]. The same was seen for the use of renal replacement therapy (RRT) [8–11]. Recent meta-analyses show rates between 29 and 33% for the occurrence of AKI [12, 13].

As in non-COVID-19 patients, AKI is associated with increased mortality in critically ill COVID-19 patients [4, 14–16].

While renal tropism of SARS-CoV-2 on the kidney was presumed to be the primary cause at the beginning of the pandemic [17], histological findings suggest a multifactorial etiology of AKI in COVID-19 patients [18]. Since COVID-19 in critically ill patients affects mostly the lungs, proposed lung-kidney interactions may be of particular importance [19]. Invasive mechanical ventilation (IMV) [20], as well as the acute respiratory distress syndrome (ARDS) [21] are known risk factors for the development of AKI in critically ill patients. During the first wave, IMV was used predominantly in critically ill COVID-19 patients due to reports from China of rapid deterioration [15, 22]. Over the course of the pandemic, the rates of IMV declined and non-invasive ventilation (NIV) strategies became more widespread. Associated with that, several studies reported a significant drop in AKI rates [23, 24].

At the beginning of the SARS-CoV-2 pandemic a registry study was established in Tyrol, Austria, which included all ICUs treating critically ill COVID-19 patients [25]. This covers a region with about 750,000 inhabitants.

The aim of this study was to investigate the incidence of, and the risk factors for, AKI in critically ill COVID-19 patients and its association with mortality in this cross-regional registry in Tyrol, Austria.

## Methods

### Patients

This prospective, multicenter registry study includes all critically ill COVID-19 patients admitted to an ICU in Tyrol (i.e., 13 departments allocated in 8 hospitals; a detailed list of all sites is available in the electronic supplemental material, [ESM] Supplemental Table 1) in the period from 1st February, 2020 until 1st May, 2022 [24, 25]. Patients required at least one positive SARS-CoV-2 by polymerase chain reaction (PCR) test for COVID-19 diagnosis. This registry was approved by the local ethics committee (Nr. 1099/2020).

Since SARS-CoV-2 spread episodically in Austria, four waves were defined as follows: first wave: 1st February, 2020 to 16th July, 2020; second wave: 17th July, 2020 to 21st February, 2021; third wave: 22nd February, 2021 to 19th July, 2021; fourth wave: 20th July 2021, to 1st May, 2022 (ESM: Supplemental Figure 1).

The detailed methods of this registry have been published previously [25].

### Definitions and data collection

Only adult patients (≥ 18 years) were included in this analysis. Endotracheal intubation and ventilation via tracheostomy were classified as IMV, while nasal high flow (NHF) and continuous positive airway pressure (CPAP) conducted by mask or helmet were categorized as non-invasive ventilation.

The occurrence of AKI during the ICU stay was defined by the Kidney Disease: Improving Global Outcomes (KDIGO) guidelines as increased serum creatinine or decreased urine output [26]. In all participating ICUs only continuous forms of RRT were provided. Depending on the ICU, these were continuous venovenous hemofiltration, continuous venovenous hemodialysis or continuous venovenous hemodiafiltration. When the modality was used for at least 2 h in a day it was counted as a day.

In case of ICU-to-ICU transfer, both the initial ICU stay and the subsequent ICU stay were combined and considered as one admission. At the time of ICU admission, the Sequential Organ Failure Assessment Score (SOFA) [27] and Simplified Acute Physiology Score 3 (SAPS 3) [28, 29] were calculated.

Baseline characteristics were extracted from the patient information system and recorded in the Tyrol-CoV-ICU-Reg. A predefined set of comorbidities was checked for every patient as detailed in the supplementary material. Intensive care interventions like IMV, NIV, prone positioning, pharmacological hemodynamic support, RRT, ECMO, duration of interventions and medication, as well as complications like AKI were recorded.

Data were collected until death or hospital discharge, whichever occurred earlier.

All data were collected with an electronic case report form and REDCap electronic data capture, a web platform for managing databases and surveys created by Vanderbilt University and hosted by the Department of Medical Statistics, Information and Health Economics, Medical University Innsbruck [30, 31].

### Statistical analysis

Median with interquartile range (IQR) is presented for continuous variables, while categorical variables are described as numbers with corresponding percentages. Adjusted odds ratio (OR) is reported with 95% confidence interval (95% CI).

For statistical analyses we used the software SPSS (version 27; IBM Corp., Armonk, New York) and R software (version 3.4.0, R Foundation for Statistical Computing, Vienna, Austria). To test for normal distribution, we performed the Shapiro–Wilk test. *T*-test was applied in case of normal distribution, otherwise the Mann–Whitney-*U*-test or Kruskal–Wallis test was performed. Categorical variables were analyzed by calculating the χ^2^-test.

We compared the number of observed deaths to the number of expected deaths for each KDIGO stage as predicted by the SAPS 3 and calculated the ratio of observed-to-expected deaths for each stage. The 95% CIs for observed deaths were calculated by bootstrapping. The Hosmer and Lemeshow test was used to test the goodness of fit of the SAPS 3 at each KDIGO stage [32].

Potential predictors for AKI and hospital mortality were evaluated using univariable and multivariable logistic regression analysis. To further identify predictors of hospital mortality a Cox regression was performed. The variables were selected based on univariate analysis. For the Cox regression analysis, we assumed that most AKI episodes occurred during the first 48 h after ICU admission.

All variables with a *p*-value < 0.05 in the univariable analysis were included in the multivariable analysis.

Statistical significance was defined as a *p*-value < 0.05 for all tests. All tests were 2-sided.

## Results

### Study population and patient characteristics

In total, 1059 patients were included during the study period in the Tyrol-CoV-ICU-Reg study. After exclusion of 17 patients < 18 years, 1042 patients were included in the final analysis. Baseline characteristics and scores at admission are reported in Table 1. The median age of the overall cohort was 66 years (IQR, 56–75) and the most common comorbidities were hypertension (57.6%), cardiovascular diseases (37.1%), obesity (Body Mass Index [BMI] > 30; 32.1%), diabetes mellitus type 2 (23.3%) and chronic kidney disease (CKD, 18.9%). At ICU admission the median SAPS 3 and SOFA score was 53 (IQR, 46–61), and 5 (IQR, 4–7), respectively. The median time from symptom onset to hospital and ICU admission was 6 days (IQR, 4–9) and 8 days (IQR, 5–11), respectively.

**Table 1:** Baseline characteristics

	Overall (*n* = 1042)	No AKI (*n* = 761)	AKI (*n* = 267)	*p*
Sex (male, *n*, %)	710 (68.1)	507 (66.6)	193 (72.3)	0.088
Age (median, IQR)	66.0 (56.0–75.0)	65.0 (54.0–74.0)	70.0 (59.0–78.0)	< 0.001
Age group (*n*, %)				
< 40	62 (6.0)	56 (7.4)	6 (2.2)	< 0.001
40–60	297 (28.5)	231 (30.4)	65 (24.3)	
60–80	558 (53.6)	398 (52.3)	152 (56.9)	
> 80	125 (12.0)	76 (10.0)	44 (16.5)	
SOFA Score (median, IQR)	5.0 (4.0–7.0)	4.0 (3.0–6.0)	7.0 (4.0–9.0)	< 0.001
SAPS 3 (median, IQR)	53.0 (46.0–61.0)	51.0 (46.0–60.0)	58.0 (51.0–65.0)	< 0.001
Comorbidities				
Hypertension (*n*, %)	600 (57.6)	406 (53.4)	186 (69.7)	< 0.001
Cardiovascular (*n*, %)	387 (37.1)	252 (33.1)	131 (49.1)	< 0.001
Obesity (BMI > 30) (*n*, %)	322 (32.1)	239 (32.7)	82 (31.4)	0.695
Diabetes mellitus (*n*, %)				
No Diabetes	729 (70.4)	532 (70.4)	185 (69.8)	0.830
DM Type 1	10 (1.0)	8 (1.1)	2 (0.8)	
DM Type 2	241 (23.3)	174 (23.0)	66 (24.9)	
Prediabetes	55 (5.3)	42 (5.6)	12 (4.5)	
Chronic kidney disease (*n*, %)	197 (18.9)	110 (14.5)	83 (31.1)	< 0.001
Hepatic (*n*, %)	88 (8.4)	56 (7.4)	31 (11.6)	0.032
Hematological malignancy (*n*, %)	53 (5.1)	33 (4.3)	18 (6.7)	0.119
Non hematological malignancy (*n*, %)	73 (7.0)	51 (6.7)	20 (7.5)	0.666
Immunosuppression (*n*, %)	73 (7.0)	47 (6.2)	25 (9.4)	0.079
COPD (*n*, %)	129 (12.4)	89 (11.7)	40 (15.0)	0.163
Asthma (*n*, %)	45 (4.3)	34 (4.5)	11 (4.1)	0.811
Other respiratory (n, %)	77 (7.4)	50 (6.6)	27 (10.1)	0.059
No known comorbidity (*n*, %)	138 (13.8)	122 (16.8)	14 (5.4)	< 0.001
Symptom onset to hospitalization (median, IQR)	6 (4–9)	6 (4–9)	6 (3–9)	0.704
Symptom onset to ICU admission (median, IQR)	8 (5–11)	9 (6–11)	8 (5–11)	0.055

*SOFA* sequential organ failure assessment, *SAPS 3* simplified acute physiology score 3, *DM* diabetes mellitus, *COPD* chronic obstructive pulmonary disease, *ICU* intensive care unit

### Acute kidney injury

Of the included patients, 267 (25.6%) developed AKI during the ICU stay. According to the KDIGO classification 8.1% had AKI stage 1, 4.9% had AKI stage 2 and 12.7% had AKI stage 3.

Patients with AKI were older and more often had a pre-existing diagnosis of hypertension, cardiovascular disease, or a known CKD. Distribution of sex and the time from symptom onset to hospital and ICU admission was similar in both (AKI and no AKI) groups (hospitalization: 6 days vs. 6 days, *p* = 0.704; ICU admission: 8 days vs. 9 days, *p* = 0.055). Compared to patients without AKI, patients with AKI had higher SOFA and SAPS 3 scores on admission (SAPS 3: 58 vs. 51, *p* < 0.001; SOFA: 7 vs. 4, *p* < 0.001) and stayed significantly longer in the ICU (19 days vs. 9 days, *p* < 0.001) and in the hospital (28 days vs. 20 days, *p* < 0.001). Interventions and outcome of patients classified according to their KDIGO stage are reported in the ESM (Supplemental Table 2). In total, 126 patients (12.3%) required RRT with a median duration of 9 days (IQR, 3–18).

### Outcome

**Fig. 1:**
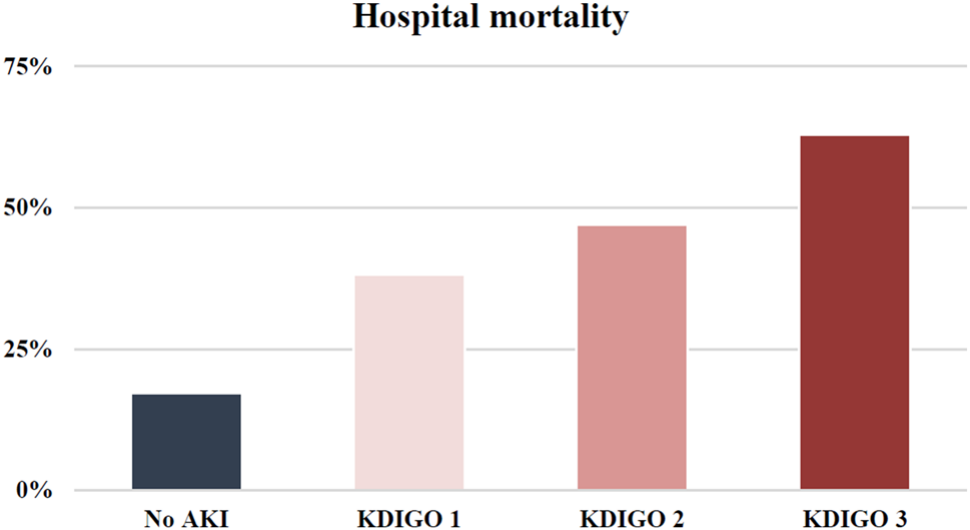
Acute kidney injury classified according to KDIGO and hospital mortality

Overall hospital and ICU mortality rates in our cohort were 26.4% and 23.1%, respectively (Table 2). In patients with AKI these rates were significantly higher (hospital mortality: 52.1% vs. 17.2%, *p* < 0.001, ICU-mortality: 47.2% vs. 14.7%, *p* < 0.001) and increased for each KDIGO stage (Fig. 1).

**Table 2:** Treatment, outcome and treatment limitations

	Overall (*n* = 1042)	No AKI (*n* = 761)	AKI (*n* = 267)	*p*
Treatment				
IMV (*n*, %)	545 (52.4)	312 (41.0)	227 (85.0)	< 0.001
Prone Positioning (*n*, %)	528 (51.0)	322 (42.5)	199 (74.8)	< 0.001
Vasopressors (*n*, %)	544 (52.7)	313 (41.3)	229 (86.4)	< 0.001
Corticosteroids (*n*, %)	840 (83.9)	618 (83.4)	209 (85.0)	0.565
RRT (*n*, %)	126 (12.3)	16 (2.1)	110 (41.4)	< 0.001
ECMO (*n*, %)				
vv-ECMO	37 (3.6)	19 (2.5)	18 (6.8)	< 0.001
va-ECMO	3 (0.3)	0	3 (1.1)	< 0.001
Days IMV (median, IQR)	13 (7–23)	12 (7–20)	16 (9–29)	0.003
Days NIV (median, IQR)	4 (1–7)	4 (2–7)	2 (1–6)	< 0.001
Days NHF (median, IQR)	3 (1–7)	4 (2–7)	2 (1–4)	< 0.001
Days Prone Positioning (median, IQR)	3 (1–6)	3 (1–5)	4 (2–7)	0.050
Days RRT (median, IQR)	9 (3–18)	9 (1–14)	9 (3–19)	0.997
Days ECMO (median, IQR)	21 (12–30)	25 (14–32)	20 (11–28)	0.763
Antiviral Drugs (*n*, %)	244 (23.8)	198 (26.1)	46 (17.2)	0.004
Remdesivir (*n*, %)	99 (9.6)	76 (10.0)	23 (8.6)	0.513
Corticosteroids (*n*, %)	827 (83.8)	618 (83.4)	209 (85.0)	0.565
Outcome				
ICU death (*n*, %)	241 (23.1)	112 (14.7)	126 (47.2)	< 0.001
Hospital death (*n*, %)	275 (26.4)	131 (17.2)	139 (52.1)	< 0.001
Length of hospital stay (median, IQR)	21 (13–36)	20 (13–32)	28 (15–48)	< 0.001
Length of ICU stay(median, IQR)	11 (5–22)	9 (4–17)	19 (9–33)	< 0.001
Treatment limitations				
DNR (*n*, %)	183 (17.6)	105 (13.8)	74 (27.7)	< 0.001
No further intervention (*n*, %)	102 (9.8)	72 (9.5)	26 (9.7)	0.895
No ECMO (*n*, %)	148 (14.2)	81 (10.6)	64 (24.0)	< 0.001
BSC (*n*, %)	116 (11.1)	50 (6.6)	65 (24.3)	< 0.001

*IMV* invasive mechanical ventilation, *RRT* renal replacement therapy, *NIV* non-invasive ventilation, *NHF* nasal high flow, *ECMO* extracorporeal membrane oxygenation, *ICU* intensive care unit, *DNR* do not resuscitate, *BSC* best supportive care

After adjustment for confounding variables such as age, IMV, vasopressor use, SAPS 3 and comorbidities (cardiovascular, hypertension and CKD), AKI KDIGO stage 2 and stage 3 were both independent predictors of hospital mortality in a logistic regression analysis (Table 3).

**Table 3:** Logistic regression analysis for prediction of hospital mortality

		Univariable		*p*	Multivariable		*p*
		OR	95% CI		OR	95% CI	
Hospital mortality	Female	0.86	0.64–1.16	0.318			
	Age	1.07	1.05–1.08	** < 0.001**	1.05	1.03–1.07	** < 0.001**
	HbA1c%	1.07	0.95–1.21	0.291			
	IMV	2.96	2.20–3.99	** < 0.001**	1.43	0.85–2.40	0.175
	Vasopressor use	3.69	2.71–5.02	** < 0.001**	1.57	0.94–2.62	0.086
	Cardiovascular	2.63	1.98–3.49	** < 0.001**	1.19	0.82–1.73	0.365
	Hypertension	1.95	1.45–2.61	** < 0.001**	1.04	0.71–1.52	0.837
	CKD	2.62	1.89–3.62	** < 0.001**	1.61	0.76–1.76	0.486
	COPD	2.09	1.43–3.06	** < 0.001**	1.59	0.98–2.59	**0.061**
	KDIGO 1	1.81	1.14–2.88	**0.012**	1.40	0.78–2.51	0.265
	KDIGO 2	2.62	1.49–4.63	** < 0.001**	2.27	1.15–4.51	**0.019**
	KDIGO 3	6.33	4.30–9.33	** < 0.001**	4.31	2.62–7.08	** < 0.001**
	SAPS 3	1.08	1.07–1.10	** < 0.001**	1.04	1.03–1.06	** < 0.001**

*IMV *invasive mechanical ventilation, *COPD* chronic obstructive pulmonary disease, *CKD* chronic kidney disease, *KDIGO *kidney disease improving global outcomes, *SAPS* simplified acute physiology score

Similar results were obtained when predictors of hospital mortality were analyzed by Cox-regression analysis (ESM Supplemental Table 3). After adjustment for confounding variables KDIGO stage 2 and stage 3 remained significant.

To account for differences regarding location we created another model, including the site of the first presentation (reference: tertiary hospital). After adjustment for confounding variables, the site of presentation was not a significant predictor for hospital mortality (ESM Supplemental Tables 4a and b). However, KDIGO stage 2 and stage 3 both remained significant after adjustment.

The frequency of AKI and the hospital mortality for each participating center is shown in ESM Supplemental Tables 5 and 6.

**Fig. 2:**
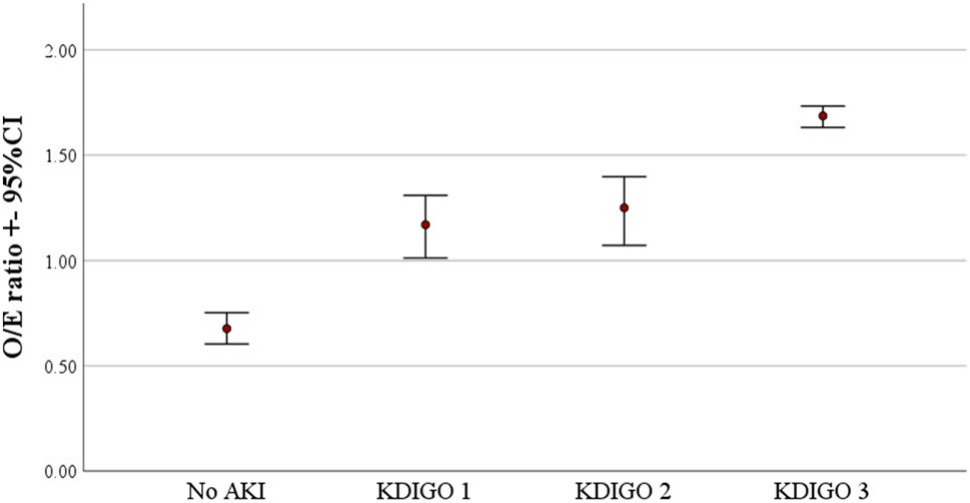
Observed to expected mortality ratios (according to the SAPS 3) of acute kidney injury classified by KDIGO and 95% confidence intervals. *AKI* acute kidney injury, *KDIGO* Kidney Disease: Improving Global Outcomes

Figure 2 shows the standardized mortality ratios ([SMR], i.e., observed-to-expected mortality ratios). In patients without AKI the SMR was 0.68 and increased with each KDIGO stage up to a maximum of nearly 2 in AKI KDIGO stage 3. The goodness of fit of the SAPS 3 score was 0.173 in AKI KDIGO stage 3.

### Variations over time

The study period can be divided into 4 waves according to the dynamics of the pandemic (ESM Supplemental Figure 1). In the first wave the rate of AKI was high, at 49.1% and it decreased in the subsequent waves (ESM Supplemental Figure 2). The main difference between the four waves was the rate of IMV, which was higher in the first compared to the other three waves (67.9% vs. 50.7% vs. 50.3% vs. 50.5%).

### Risk factors for AKI

Overall, 52.4% (*n* = 545) of the study population received IMV during the ICU stay for a median of 13 days (IQR, 7–23). In patients with AKI the IMV rate was significantly higher with 85.0% compared to 41.0% in the no AKI group. Patients with AKI required mechanical ventilation for a longer period compared to patients without AKI (16 days vs. 12 days, *p* = 0.003).

After adjusting for confounding variables, IMV and vasopressor use were significant predictors of AKI in a multivariable logistic regression analysis (Table 4).

**Table 4:** Logistic regression analysis for prediction of acute kidney injury

		Univariable			Multivariable		
		OR	95% CI	*p*	OR	95% CI	*p*
AKI	Female	0.77	0.56–1.04	0.088			
	Age	1.03	1.02–1.04	** < 0.001**	1.01	1.00–1.03	0.170
	HbA1c%	1.05	0.93–1.18	0.433			
	IMV	8.17	5.67–11.77	** < 0.001**	4.19	2.48–7.08	** < 0.001**
	Vasopressor use	9.04	6.19–13.22	** < 0.001**	3.17	1.87–5.35	** < 0.001**
	Cardiovascular	1.95	1.47–2.58	** < 0.001**	1.35	0.92–1.97	0.122
	Hypertension	2.01	1.49–2.70	** < 0.001**	1.46	1.01–2.12	**0.044**
	CKD	2.67	1.92–3.71	** < 0.001**	2.30	1.51–3.49	** < 0.001**
	COPD	1.33	0.89–1.99	0.164			
	SAPS 3	1.05	1.03–1.06	** < 0.001**	1.01	1.00–1.03	0.078

*IMV* invasive mechanical ventilation, *COPD* chronic obstructive pulmonary disease, *CKD* chronic kidney disease, *KDIGO* kidney disease improving global outcomes, *SAPS* simplified acute physiology score

## Discussion

In this prospective observational multicenter cohort study of critically ill adult COVID-19 patients we found an incidence of AKI of 25.6% and a rate of RRT of 12.3%. Patients with AKI showed significantly higher hospital mortality compared to patients without AKI. After adjustment for other important risk factors, AKI KDIGO stage 2 and stage 3 remained independent predictors of hospital mortality. The most important risk factors for AKI were IMV, vasopressor use and CKD in a multivariable logistic regression analysis. Among known risk factors, IMV seems to be of particular importance as a predictor of AKI in critically ill COVID-19 patients.

The rate of AKI in patients with confirmed SARS-CoV-2 varied in single reports at the onset of the pandemic. Meanwhile, systematic reviews and meta-analyses found a range from 29% to 37% [12, 33]. In our cohort we found a relatively low overall rate of AKI of 23%, compared to other studies [34, 35]. This may be due to differences in case-mix and baseline characteristics or treatment standards, as shown in our previous studies [24, 25].

It is well known that AKI is associated with increased mortality in critically ill non-COVID-19 patients [4]. Acute kidney injury is also an important risk factor for mortality in COVID-19 ICU patients as shown by previous investigations [36]. We observed a fourfold increased risk of death in patients with AKI KDIGO Stage 3. Even after adjustment for the most common risk factors for COVID-19, AKI KDIGO Stages 2 & 3 remained significant predictors of mortality in critically ill COVID-19 patients. A recent study indicated that AKI may contribute independently to mortality only in less severe critically ill COVID-19 patients [37]. When we corrected for disease severity by including the SAPS 3 in our model, AKI remained an independent predictor of mortality (ESM Supplemental Tables 3 and 4b).

Correspondingly, we found an increased SMR (based on the SAPS 3) with each AKI KDIGO stage. In patients without AKI the SMR mortality ratio was below 1, reflecting good performance of the participating ICUs. However, AKI appeared to be an independent factor contributing to significantly impaired outcome. KDIGO 1 was already associated with a significantly increased O/E mortality ratio of 1.17. This was further increased in KDIGO stage 2 and stage 3, contributing to a nearly twofold increased excess SMR. Similar findings have been reported for AKI in critically ill patients not suffering from COVID-19 [38].

Rates of AKI in the first wave were higher compared to the consecutive waves in our study population. Interestingly in the first wave, IMV rates were also much higher (ESM Supplemental Figure 2). One reason for the high rates of IMV observed at the beginning of the pandemic might be the tendency to resort to early intubation due to uncertainty in the management of rapid respiratory deterioration in COVID-19-related ARDS [39]. Invasive mechanical ventilation rates decreased in the subsequent waves and more patients were primarily treated with non-invasive ventilation. Accordingly, AKI rates decreased from the first to the following waves. This occurred even though patients in the second wave were significantly older than in the first wave, which even resulted in increased mortality in that period, as previously reported [24]. Since age is an established risk factor for AKI [40], this enhances the importance of these findings.

In a (pre-pandemic) meta-analysis, van den Akker et al. showed that IMV is an independent risk factor for AKI in critically ill patients [20]. The mechanisms are complex and multi-factorial. One mechanism of kidney damage from IMV is high ventilation pressures (especially positive end-expiratory pressure [PEEP]) and the resulting intrathoracic pressures. This reduces the venous return to the heart, which leads to decreased cardiac output, venous congestion and increased abdominal pressure. Consequently, these altered pressure conditions accompanied by activated neurohormonal responses may lead to a reduction in renal blood flow and glomerular filtration [19]. A small pilot study suggested that these effects may be particularly pronounced in COVID-19 compared to mechanically ventilated patients with ARDS of different etiology [41]. Exceptionally high ventilatory pressures were often required in COVID-19 ARDS patients [42], contributing to barotrauma and biotrauma of the lung associated with increased release of pro-inflammatory cytokines [43]. However, COVID-19-specific lung-kidney interactions may become effective even before mechanical ventilation is required. Pulmonary microthrombi formation, increased pulmonary resistance and central venous pressure may contribute to renal congestion and impair renal blood flow as well as renal function, as shown for other patient populations [44].

Other observational studies reporting AKI in COVID-19 found a similar association with IMV [35, 45]. In our study IMV was among the most important predictors of AKI, together with vasopressor use. However, it should be noted that these two interventions are closely interrelated, mainly because of the hemodynamic impact of sedation during IMV, and therefore it is difficult to consider their effects separately.

Another important risk factor for AKI is pre-existing kidney damage [46]. Many studies investigating AKI in COVID-19 patients identified CKD as a risk factor for AKI [47]. In our study CKD was a predictor for AKI, which is consistent with many reports independently of the pandemic [47, 48]. Reduced renal reserve leading to higher susceptibility to AKI is among the proposed mechanisms.

Treatment recommendations changed over time, especially after the publication of the RECOVERY trial on dexamethasone [49]. The impact of steroid treatment on kidney outcomes is difficult to determine since nearly all COVID-19 patients who required oxygen therapy were treated with steroids after the first wave. This may also have contributed to the lower IMV rates after the first wave. However, it is likely that a better understanding of SARS-CoV-2 and improvements in treatment also contributed to a decrease in the AKI rates. The same applies to the different emerging variants in the four waves. We cannot exclude that the different viral mutations might also have contributed to different susceptibility for AKI.

In our cohort, we found a rate of RRT in AKI patients of about 40%, which is higher than rates reported by studies performed in the pre-pandemic era [4]. This may be a COVID-19-specific finding due to the multifactorial mechanisms contributing to AKI in this population [19] influencing requirement of RRT. Other studies comparing COVID-19 to non-COVID-19 patients [9] and a recent meta-analysis [50] also found higher rates of RRT in (hospitalized) COVID-19 patients. In a sepsis cohort however, similar high rates of around 30% have been reported by Peters et al. [51].

Limitations of this study are related to its observational design. We cannot fully exclude that the higher disease severity scores in patients with IMV led to a higher rate of AKI, whereby it must be noted that both the SAPS 3 and the SOFA score considering the modality of ventilation for score calculation and therefore, IMV and vasopressor use, actually led to higher scores themselves. As a consequence of the multicenter character of this study, differences in treatment strategies among centers cannot be excluded. However, we included the location of the first presentation in an additional analysis in the supplementary material. Due to the ongoing pandemic, changes in medical care over time may also have influenced the results. Unfortunately, some important data, such as CKD stage, baseline eGFR or the time-point of the occurrence of AKI are not available for our data set.

The main strengths of this study are the inclusion of all critically ill patients treated over the first four COVID-19 waves in the whole region of Tyrol, Austria. Therefore, our study includes patients from central as well as peripheral hospitals.

## Conclusions

AKI is common in critically ill COVID-19 patients and is associated with impaired outcome. Patients with IMV have a higher risk of AKI during their ICU stay. Even when adjusting for common risk factors, IMV remains an independent predictor of AKI in critically ill COVID-19 patients. Therefore, preventive actions may be particularly important in COVID-19 patients undergoing intermittent mechanical ventilation.

### Supplementary Information

Below is the link to the electronic supplementary material.

Supplementary file1 (DOCX 256 KB)

## Data Availability

The datasets used and analysed in the current study are available from the corresponding author on reasonable request.
