# Supplementary material for: Incidence, risk factors and outcome of acute kidney injury in critically ill COVID-19 patients in Tyrol, Austria: a prospective multicenter registry study
Source: J Nephrol. 2023 Oct 14;36(9):2531–40. doi: 10.1007/s40620-023-01760-3 (PMC10703973; doi:10.1007/s40620-023-01760-3)
Supplement: Supplementary file 1 — Supplementary file1 (DOCX 256 KB) [file 40620_2023_1760_MOESM1_ESM.docx]

Electronic supplemental material

**Incidence, risk factors and outcome of acute kidney injury in critically ill COVID-19 patients in Tyrol, Austria**

**– a prospective multicenter registry study**

Supplemental Figure 1: Active cases of critically ill COVID-19 patients per day in Tyrol, Austria
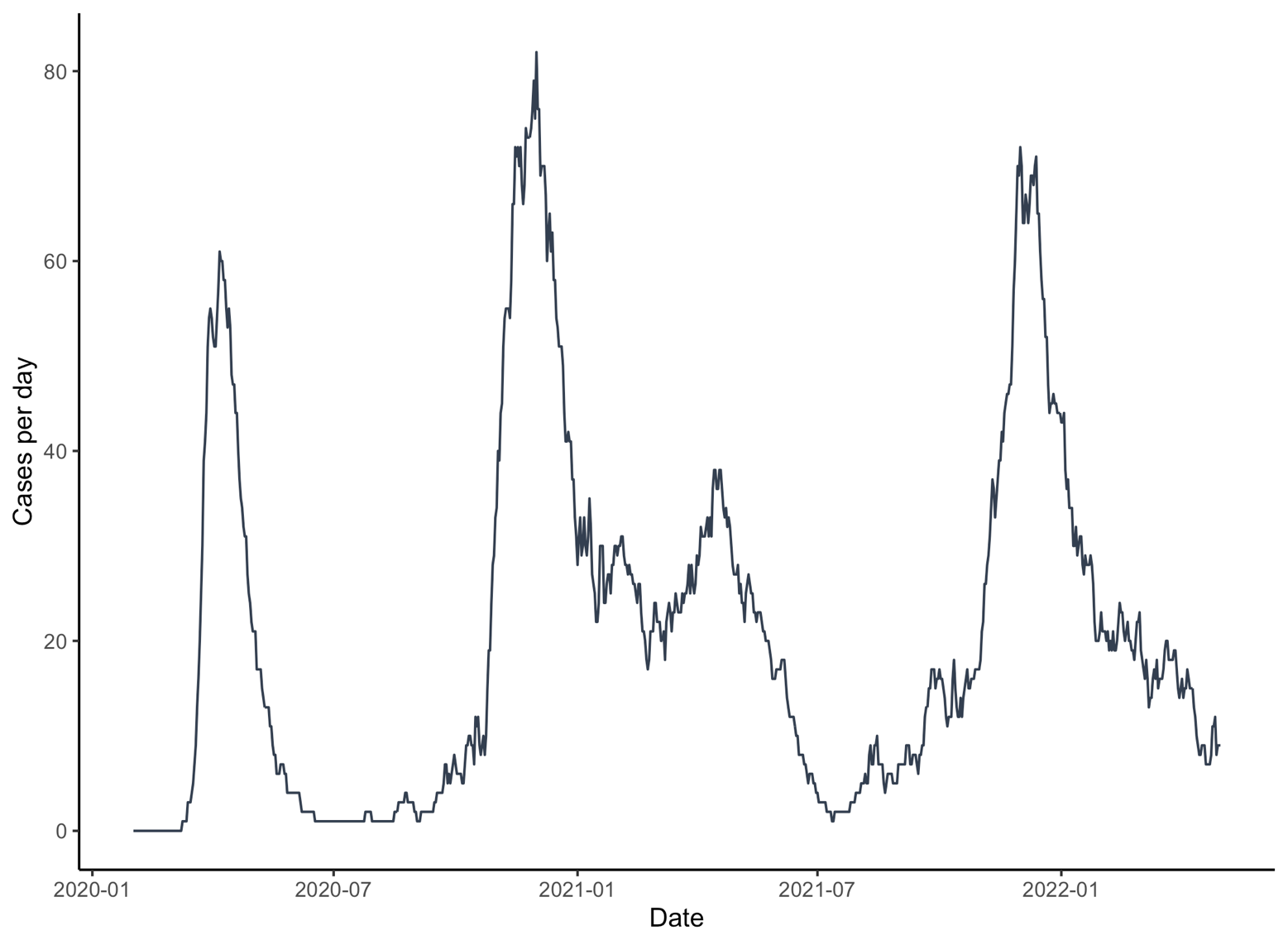


Supplemental Figure 2: Frequency of IMV, AKI, RRT and Mortality over 4 Waves


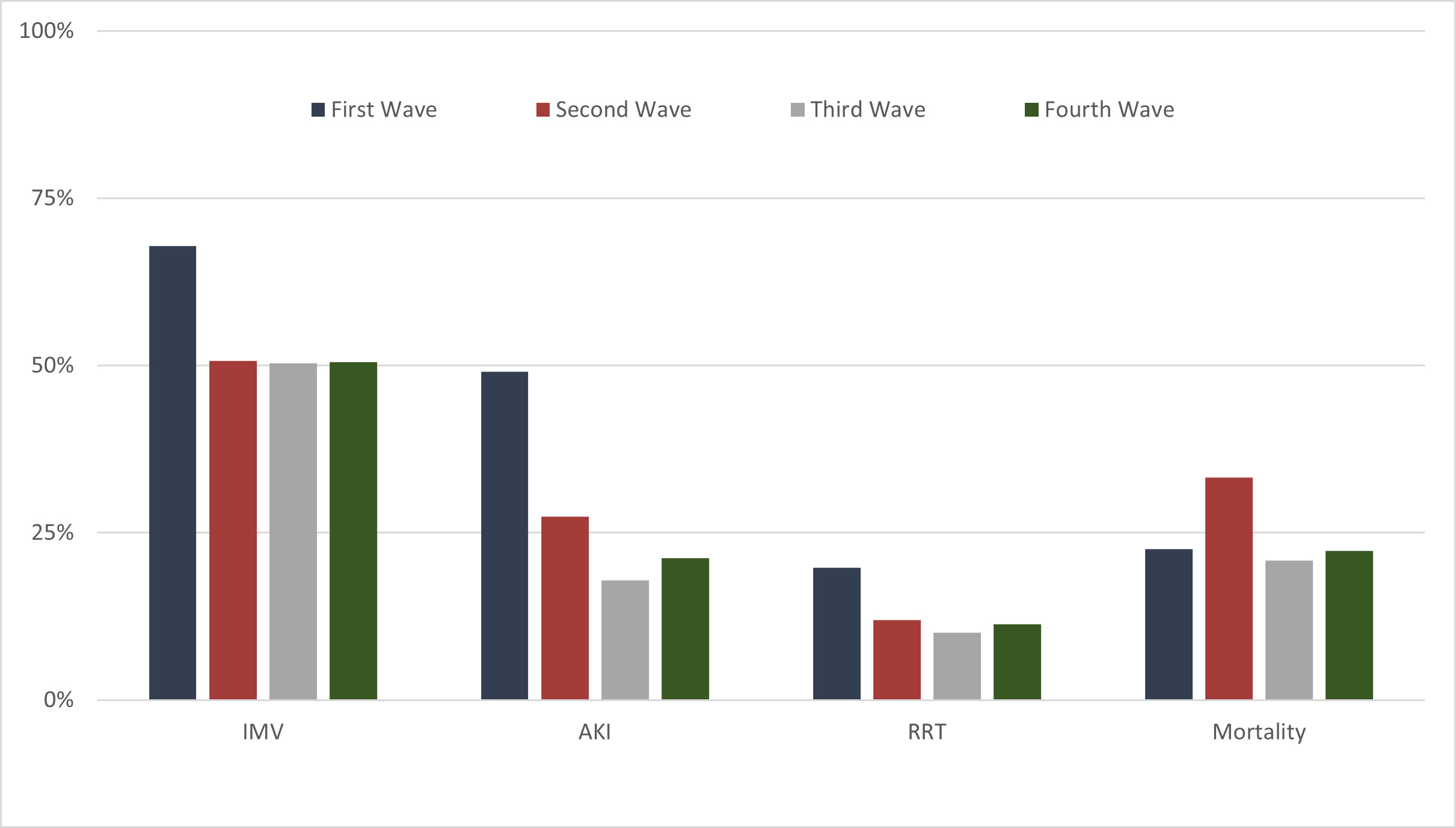
*IMV - invasive mechanical ventilation, AKI – acute kidney injury, RRT – renal replacement therapy*

Supplemental Table 1: List of COVID-19 intensive care units (ICUs) in Tyrol, Austria who participated in the Tyrolean COVID-19 Intensive Care Registry (Tyrol-CoV-ICU-Reg):

|  |  |
| --- | --- |
| Medical University Innsbruck, Innsbruck, Austria | First wave:   - Medical ICU, Department of Internal Medicine - Neurosurgical ICU, Department of Neurosurgery - Transplantation ICU, Department of General and Surgical Intensive Care Medicine/Department of Anesthesia and Critical Care Medicine - Recovery room “OZA” (adapted as temporary ICU), Department of General and Surgical Intensive Care Medicine/Department of Anesthesia and Critical Care Medicine - Recovery room “KHZ” (adapted as temporary ICU), Department of General and Surgical Intensive Care Medicine/Department of Anesthesia and Critical Care Medicine - Pediatric Intensive Care Unit, Department of Pediatrics   Second, third and fourth wave:   - Medical ICU, Department of Internal Medicine - Neurosurgical ICU, Department of Neurosurgery - Transplantation ICU, Department of General and Surgical Intensive Care Medicine/Department of Anesthesia and Critical Care Medicine - General surgery ICU, Department of General and Surgical Intensive Care Medicine/Department of Anesthesia and Critical Care Medicine - Cardiac surgery ICU, Department of General and Surgical Intensive Care Medicine/Department of Anesthesia and Critical Care Medicine - Pediatric Intensive Care Unit, Department of Pediatrics |
| Hospital Hall, Hall, Austria | ICU, Department of Anesthesia and Intensive Care Medicine |
| Hospital Kufstein, Kufstein, Austria | ICU, Department of Anesthesia and Intensive Care Medicine |
| Hospital Lienz, Lienz, Austria | ICU, Department of Anesthesia and Intensive Care Medicine |
| Hospital Reutte, Reutte, Austria | ICU, Department of Anesthesia and Intensive Care Medicine |
| Hospital Schwaz, Schwaz, Austria | ICU, Department of Anesthesia and Critical Care Medicine |
| Hospital St. Johann i.T., St. Johann in Tyrol, Austria | ICU, Department of Anesthesia and Intensive Care Medicine |
| Hospital Zams | Medical ICU, Department of Internal Medicine  Surgical ICU, Department of Anesthesiology and Critical Care Medicine |

Supplemental Table 2: Treatment, Outcome and Treatment limitations

|  |  | No AKI  (n = 761) | KDIGO 1  (n = 84) | KDIGO 2  (n = 51) | KDIGO 3  (n = 132) | p |
| --- | --- | --- | --- | --- | --- | --- |
| **Treatment** |  |  |  |  |  |  |
| IMV (n, %) |  | 312 (41.0%) | 66 (78.6%) | 41 (80.4%) | 120 (90.9%) | <.001 |
| Prone Positioning (n, %) |  | 322 (42.5%) | 63 (75.9%) | 39 (76.5%) | 97 (73.5%) | <.001 |
| Vasopressors (n, %) |  | 313 (41.3%) | 66 (78.6%) | 42 (82.4%) | 121 (93.1%) | <.001 |
| Corticosteroids (n, %) |  | 618 (83.4%) | 68 (88.3%) | 36 (75.0%) | 105 (86.8%) | .190 |
| RRT (n, %) |  | 16 (2.1%) | 3 (3.6%) | 5 (9.8%) | 102 (77.3%) | <.001 |
| ECMO (n, %) | vv-ECMO | 19 (2.5%) | 3 (3.6%) | 2 (3.9%) | 13 (9.9%) | <.001 |
|  | va-ECMO | 0 | 1 (1.2%) | 0 | 2 (1.5%) |  |
| Days IMV (median, IQR) |  | 12 (7 – 20) | 12 (9 – 23) | 14 (9 – 23) | 17 (10 – 34) | <.001 |
| Days NIV (median, IQR) |  | 4 (2 – 7) | 4 (1 – 7) | 2 (1 – 6) | 2 (1 – 3) | <.001 |
| Days NHF (median, IQR) |  | 4 (2 – 7) | 2 (1 – 6) | 2 (1 – 4) | 2 (1 – 3) | <.001 |
| Days Prone Positioning (median, IQR) |  | 3 (1 – 5) | 3 (2 – 6) | 4 (1 – 5) | 4 (2 – 9) | .046 |
| Days RRT (median, IQR) |  | 9 (1 – 14) | 1 ( 1 – 1) | 3 (3 – 6) | 10 (3 – 21) | .009 |
| Days ECMO (median, IQR) |  | 25 (14 – 32) | 12 (6 – 19) | 18 (15 – 20) | 27 (11 – 33) | .392 |
|  |  |  |  |  |  |  |
| **Outcome** |  |  |  |  |  |  |
| ICU death (n, %) |  | 112 (14.7%) | 30 (35.7%) | 23 (45.1%) | 73 (55.3%) | <.001 |
| Hospital death (n, %) |  | 131 (17.2%) | 32 (38.1%) | 24 (47.1%) | 83 (62.9%) | <.001 |
| Length of stay hospital (median, IQR) |  | 20 (13 – 32) | 25 (16 – 35) | 24 (11 – 39) | 34 (15 – 63) | <.001 |
| Length of stay ICU (median, IQR) |  | 9 (4 – 17) | 17 (10 – 26) | 18 (7 – 30) | 22 (10 – 38) | <.001 |
|  |  |  |  |  |  |  |
| **Treatment limitation** |  |  |  |  |  |  |
| DNR (n, %) |  | 105 (13.8%) | 19 (22.6%) | 15 (29.4%) | 40 (30.3%) | <.001 |
| No further intervention (n, %) |  | 72 (9.5%) | 9 (10.7%) | 6 (11.8%) | 11 (8.3%) | .884 |
| No ECMO (n, %) |  | 81 (10.6%) | 15 (17.9%) | 10 (19.6%) | 39 (29.5%) | <.001 |
| BSC (n, %) |  | 50 (6.6%) | 16 (19.0%) | 10 (19.6%) | 39 (29.5%) | <.001 |

*IMV - invasive mechanical ventilation, RRT – renal replacement therapy, NIV – non-invasive ventilation, NHF – nasal high flow, ECMO - extracorporeal membrane oxygenation, ICU - intensive care unit, DNR – do not resuscitate, BSC – best supportive care.*

Supplemental Table 3: Cox-regression analysis for prediction of hospital mortality

|  |  | Univariate | |  | Multivariate | |  |
| --- | --- | --- | --- | --- | --- | --- | --- |
|  |  | HR | 95% CI | p | HR | 95% CI | p |
| Hospital Mortality | Female | 0.96 | 0.74 – 1.25 | .775 |  |  |  |
|  | Age | 1.06 | 1.05 – 1.08 | **<.001** | 1.04 | 1.03 – 1.06 | **<.001** |
|  | HbA1c% | 1.03 | 0.93 – 1.15 | .510 |  |  |  |
|  | Cardiovascular | 2.25 | 1.77 – 2.85 | **<.001** | 1.18 | 0.90 – 1.55 | .223 |
|  | Hypertension | 1.61 | 1.24 – 2.07 | **<.001** | 1.00 | 0.75 – 1.33 | .986 |
|  | CKD | 1.82 | 1.41 – 2.35 | **<.001** | 1.07 | 0.80 – 1.42 | .657 |
|  | COPD | 1.74 | 1.28 – 2.35 | **<.001** | 1.03 | 0.94 – 1.82 | .118 |
|  | KDIGO 1 | 1.40 | 0.97 – 2.03 | .073 | 1.24 | 0.81 – 1.89 | .330 |
|  | KDIGO 2 | 1.84 | 1.21 – 2.80 | .004 | 2.02 | 1.28 – 3.20 | **.003** |
|  | KDIGO 3 | 2.13 | 1.64 – 2.78 | **<.001** | 2.07 | 1.54 – 2.78 | **<.001** |
|  | SAPS 3 | 1.06 | 1.05 – 1.07 | **<.001** | 1.04 | 1.03 – 1.05 | **<.001** |
|  | Tertiary hospital at first presentation | 0.65 | 0.51 – 0.83 | **<.001** | 0.95 | 0.73 – 1.24 | .716 |

*HR – hazard ratio, CKD – chronic kidney disease, COPD - chronic obstructive pulmonary disease,* *KDIGO - kidney disease improving global outcomes,* *SAPS - simplified acute physiology score,*

Supplemental Table 4a: Logistic regression analysis for prediction of AKI and hospital mortality including SAPS 3 and the site of first presentation

|  |  | Univariate | |  | Multivariate | |  |
| --- | --- | --- | --- | --- | --- | --- | --- |
|  |  | OR | 95% CI | p | OR | 95% CI | p |
| AKI | Sex:Female | 0.77 | 0.56 – 1.04 | .088 |  |  |  |
|  | Age | 1.03 | 1.02 – 1.04 | **<.001** | 1.01 | 0.99 – 1.03 | .227 |
|  | HbA1c% | 1.05 | 0.93 – 1.18 | .433 |  |  |  |
|  | IMV | 8.17 | 5.67 – 11.77 | **<.001** | 4.24 | 2.50 – 7.20 | **<.001** |
|  | Vasopressors | 9.04 | 6.19 – 13.22 | **<.001** | 3.17 | 1.87 – 5.37 | **<.001** |
|  | Cardiovascular | 1.95 | 1.47 – 2.58 | **<.001** | 1.38 | 0.94 – 2.03 | .096 |
|  | Hypertension | 2.01 | 1.49 – 2.70 | **<.001** | 1.39 | 0.96 – 2.03 | .086 |
|  | CKD | 2.67 | 1.92 – 3.71 | **<.001** | 2.41 | 1.58 – 3.68 | **<.001** |
|  | COPD | 1.33 | 0.89 – 1.99 | .164 |  |  |  |
|  | SAPS 3 | 1.05 | 1.03 – 1.06 | **<.001** | 1.01 | 1.00 – 1.03 | .130 |
|  | Tertiary hospital at first presentation | 0.65 | 0.49 – 0.86 | **. 003** | 0.69 | 0.49 – 0.98 | **.036** |

*OR – odds ratio, IMV - invasive mechanical ventilation,* *CKD – chronic kidney disease, COPD - chronic obstructive pulmonary disease*

Supplemental Table 4b: Logistic regression analysis for prediction of AKI and hospital mortality including SAPS 3 and the site of first presentation

|  |  | Univariate | |  | Multivariable | |  |
| --- | --- | --- | --- | --- | --- | --- | --- |
|  |  | OR | 95% CI | p | OR | 95% CI | p |
| Hospital Mortality | Sex:Female | 0.86 | 0.64 – 1.16 | .318 |  |  |  |
|  | Age | 1.07 | 1.05 – 1.08 | **<.001** | 1.05 | 1.03 – 1.07 | **<.001** |
|  | HbA1c% | 1.07 | 0.95 – 1.21 | .291 |  |  |  |
|  | IMV | 2.96 | 2.20 – 3.99 | **<.001** | 1.43 | 0.85 – 2.41 | .173 |
|  | Vasopressors | 3.69 | 2.71 – 5.02 | **<.001** | 1.57 | 0.94 – 2.62 | .086 |
|  | Cardiovascular | 2.63 | 1.98 – 3.49 | **<.001** | 1.20 | 0.82 – 1.74 | .354 |
|  | Hypertension | 1.95 | 1.45 – 2.61 | **<.001** | 1.03 | 0.70 – 1.51 | .875 |
|  | CKD | 2.62 | 1.89 – 3.62 | **<.001** | 1.17 | 0.77 – 1.78 | .472 |
|  | COPD | 2.09 | 1.43 – 3.06 | **<.001** | 1.58 | 0.97 – 2.58 | .064 |
|  | KDIGO 1 | 1.81 | 1.14 – 2.88 | **.012** | 1.38 | 0.76 – 2.48 | .286 |
|  | KDIGO 2 | 2.62 | 1.49 – 4.63 | **<.001** | 2.26 | 1.14 – 4.49 | **.020** |
|  | KDIGO 3 | 6.33 | 4.30 – 9.33 | **<.001** | 4.31 | 2.62 – 7.08 | **<.001** |
|  | SAPS 3 | 0.92 | 0.91 – 0.94 | **<.001** | 1.04 | 1.03 – 1.06 | **<.001** |
|  | Tertiary hospital at first presentation | 0.70 | 0.53 – 0.93 | **.011** | 0.94 | 0.66 – 1.33 | .707 |

*OR – odds ratio, IMV - invasive mechanical ventilation, CKD – chronic kidney disease, COPD - chronic obstructive pulmonary disease*

Supplemental Table 5: Frequency of acute kidney injury and hospital mortality per participating centre

|  | AKI (n = 267) | Hospital Mortality (n = 275) |
| --- | --- | --- |
| Centre 1 | 41 (15.4%) | 45 (16.7%) |
| Centre 2 | 11 (39.3%) | 7 (25.0%) |
| Centre 3 | 35 (27.1%) | 37 (28.7%) |
| Centre 4 | 1 (100%) | 1 (100%) |
| Centre 5 | 2 (25.0%) | 3 (37.5%) |
| Centre 6 | 18 (27.7%) | 21 (31.8%) |
| Centre 7 | 23 (32.9%) | 15 (21.1%) |
| Centre 8 | 12 (11.9%) | 26 (25.2%) |
| Centre 9 | 30 (46.2%) | 23 (34.8%) |
| Centre 10 | 42 (38.2%) | 41 (36.6%) |
| Centre 11 | 22 (36.1%) | 22 (34.4%) |
| Centre 12 | 22 (24.2%) | 30 (32.6%) |
| Centre 13 | 5 (20.0%) | 4 (16.0%) |
| Centre 14 | 3 (75.0%) | 0 |

*AKI – acute kidney injury*

Supplemental Table 6: Frequency of acute kidney injury and hospital mortality according to the site of the first presentation

|  | AKI (n = 267) | Hospital Mortality (n = 275) |
| --- | --- | --- |
| Tertiary hospital | 108 (21.7%) | 114 (29.8%) |
| Secondary hospital | 159 (29.9%) | 161 (22.8%) |
| *AKI – acute kidney injury* |  |  |
